# Deep learning–based quantification of CT changes after 1 year of pirfenidone treatment as a prognostic marker in idiopathic pulmonary fibrosis

**DOI:** 10.1186/s13244-026-02447-6

**Published:** 2026-11-03

**Authors:** Ji Young Lee, Kum Ju Chae, Myoung-jin Jang, Jonathan H. Chung, Jin Mo Goo, Sun Mi Choi, Jimyung Park, Jong Hyuk Lee

**Affiliations:** 1 Department of Radiology, Seoul National University Hospital, Seoul, Korea; 2 Department of Radiology, Seoul National University College of Medicine, Seoul, Korea; 3 Department of Radiology, Research Institute of Clinical Medicine of Jeonbuk National University Biomedical Research Institute of Jeonbuk National University Hospital, Jeonbuk National University and Medical School, Jeonju, Korea; 4 Medical Research Collaborating Center, Seoul National University Hospital, Seoul, Korea; 5 Department of Radiology, The University of Chicago, Chicago, IL USA; 6 Institute of Radiation Medicine, Seoul National University Medical Research Center, Seoul, Korea; 7 Cancer Research Institute, Seoul National University, Seoul, Korea; 8 Division of Pulmonary and Critical Care Medicine, Department of Internal Medicine, Seoul National University Hospital, Seoul, Korea

**Keywords:** Idiopathic pulmonary fibrosis, CT, Deep learning, Prognosis

## Abstract

**Objectives:**

To evaluate the prognostic value of changes in deep learning–based CT quantification following 1 year of pirfenidone treatment in patients with idiopathic pulmonary fibrosis (IPF).

**Materials and methods:**

This study included patients with IPF who received at least 1 year of pirfenidone between September 2009 and March 2022 at two hospitals. The extent of normal lung and fibrosis (sum of reticular opacity and honeycombing) was quantified on baseline and 1-year follow-up chest CT scans using a deep learning–based tool, and the changes were calculated. The primary outcome was a composite of all-cause mortality or lung transplantation; the secondary outcome was a ≥ 10% absolute decline in forced vital capacity (FVC) occurring after the 1-year follow-up. Associations between changes in CT quantification and these outcomes were assessed using Cox proportional hazards regression analyses.

**Results:**

A total of 436 patients (341 men; mean age, 69.8 years) were included. Greater preservation of normal lung extent over 1 year was associated with a lower risk of the composite outcome (adjusted hazard ratio (HR) per 5% increase: 0.90; 95% confidence interval (CI): 0.85, 0.96; *p* < 0.001), whereas increases in fibrosis extent were associated with a higher risk (adjusted HR per 5% increase: 1.16, 95% CI: 1.06, 1.27, *p* = 0.002). Regarding the secondary outcome of ≥ 10% absolute FVC decline, only the change in fibrosis extent remained a significant predictor (adjusted HR per 5% increase: 1.26, 95% CI: 1.09, 1.45, *p* = 0.001).

**Conclusions:**

Changes in deep learning–based CT quantification results over 1 year of pirfenidone treatment demonstrated significant prognostic value in patients with IPF.

**Key Points:**

***Question*** Disease progression and mortality in patients with idiopathic pulmonary fibrosis (IPF) continue to occur unpredictably beyond 1 year of pirfenidone treatment.

***Findings*** Changes in deep learning–based quantification of normal lung and fibrosis extent after 1 year of pirfenidone treatment were significant prognostic predictors.

***Critical relevance statement*** Incorporating 1-year changes in deep learning–based CT quantification improves prognostication of mortality, lung transplantation, and lung function decline, supporting more individualized risk stratification in idiopathic pulmonary fibrosis.

**Graphical Abstract:**

**Figure:**
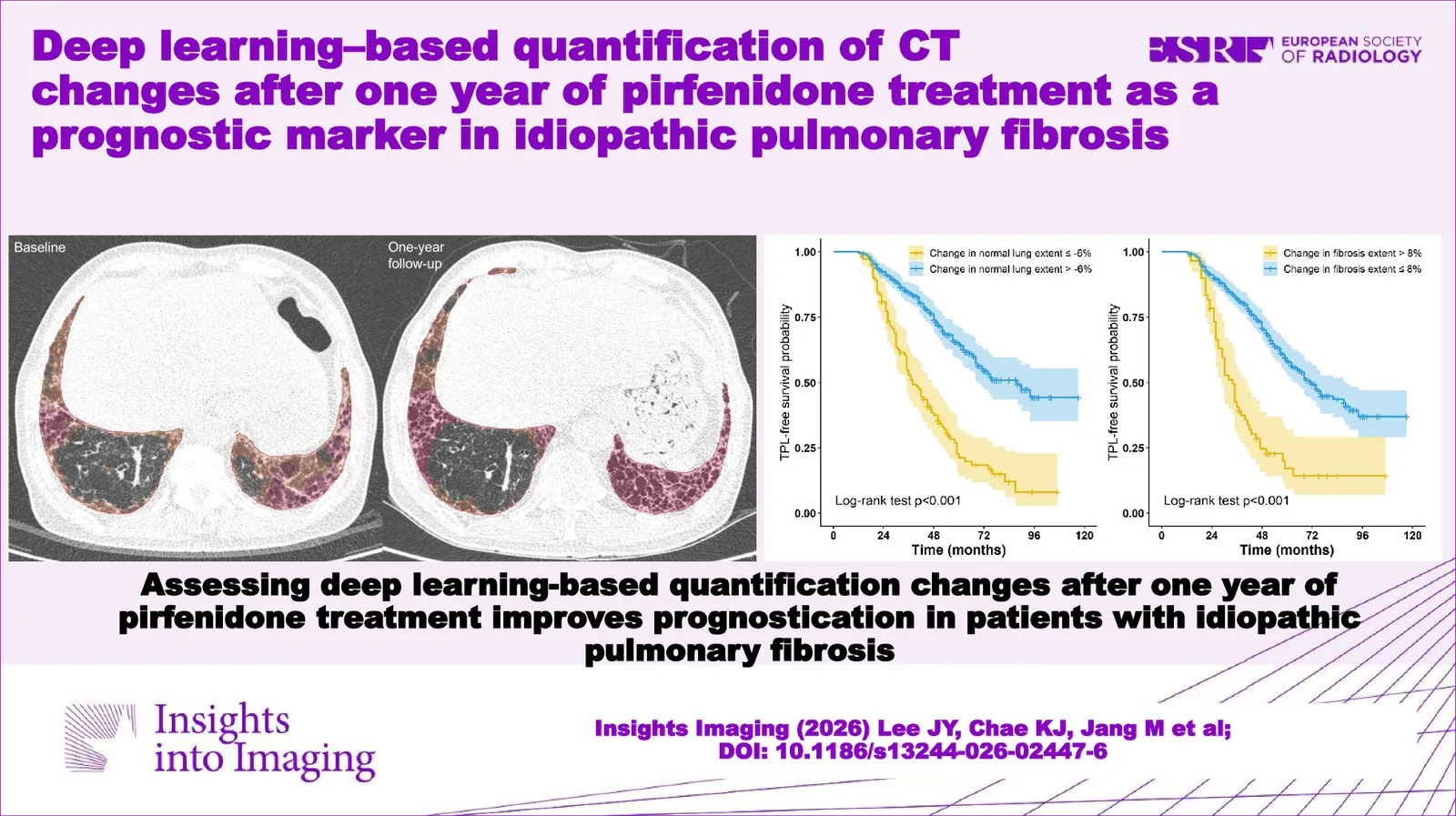

## Introduction

Idiopathic pulmonary fibrosis (IPF) is a chronic, progressive, and fatal lung disease characterized by irreversible loss of lung function and worsening dyspnea [1–3]. The natural course of IPF is heterogeneous and unpredictable; nonetheless, lung function eventually declines, with an annual rate of decline in forced vital capacity (FVC) ranging from 150 to 200 mL [1–4]. The prognosis of the disease is poor, with a median survival of 2–5 years [5–7].

Antifibrotic agents, including pirfenidone and nintedanib, play a crucial role in the treatment of IPF, as they can slow the rate of FVC decline and reduce all-cause mortality [1–3, 8]. Although landmark trials of current antifibrotic agents have focused on FVC decline over a 1-year period, the consistent occurrence of disease progression and patient death throughout the natural course of IPF necessitates research on longer-term clinical outcomes after starting antifibrotic therapy [9–12]. However, predicting the long-term prognosis in patients with IPF remains challenging due to substantial variability in the disease’s clinical course [13]. Relying solely on FVC trajectory—widely regarded as a key prognostic indicator—has proven insufficient for accurately capturing overall disease progression [14].

A potential solution to this unmet need is the use of high-resolution CT scans, which patients with IPF routinely undergo at baseline and during follow-up. High-resolution CT is integral for the diagnosis, longitudinal evaluation, and prognostication of IPF [1, 15, 16]. Recently, deep learning–based CT analysis has been introduced in IPF research [17–22], particularly for quantitative assessment, which has been reported to overcome inter- and intra-observer variability in visual CT interpretation [23–26]. In this study, we assessed the prognostic value of deep learning–based CT quantification—focusing on changes in normal lung and fibrosis extents after 1 year of treatment—for predicting all-cause mortality or lung transplantation in patients with IPF who completed at least 1 year of pirfenidone therapy.

## Materials and methods

### Study sample

This retrospective cohort study included consecutive patients diagnosed with IPF and treated with pirfenidone between September 2009 and March 2022 at two tertiary hospitals: Seoul National University Hospital and Jeonbuk National University Hospital. We included only patients who initiated treatment with pirfenidone because the number of patients starting nintedanib was extremely limited, given that nintedanib is not covered by the National Health Insurance in South Korea. This study was approved by the Institutional Review Boards of both institutions, and the requirement for written informed consent was waived (Seoul National University Hospital: H-2208-165-1354; Jeonbuk National University Hospital: CUH-2024-05-054).

**Fig. 1:**
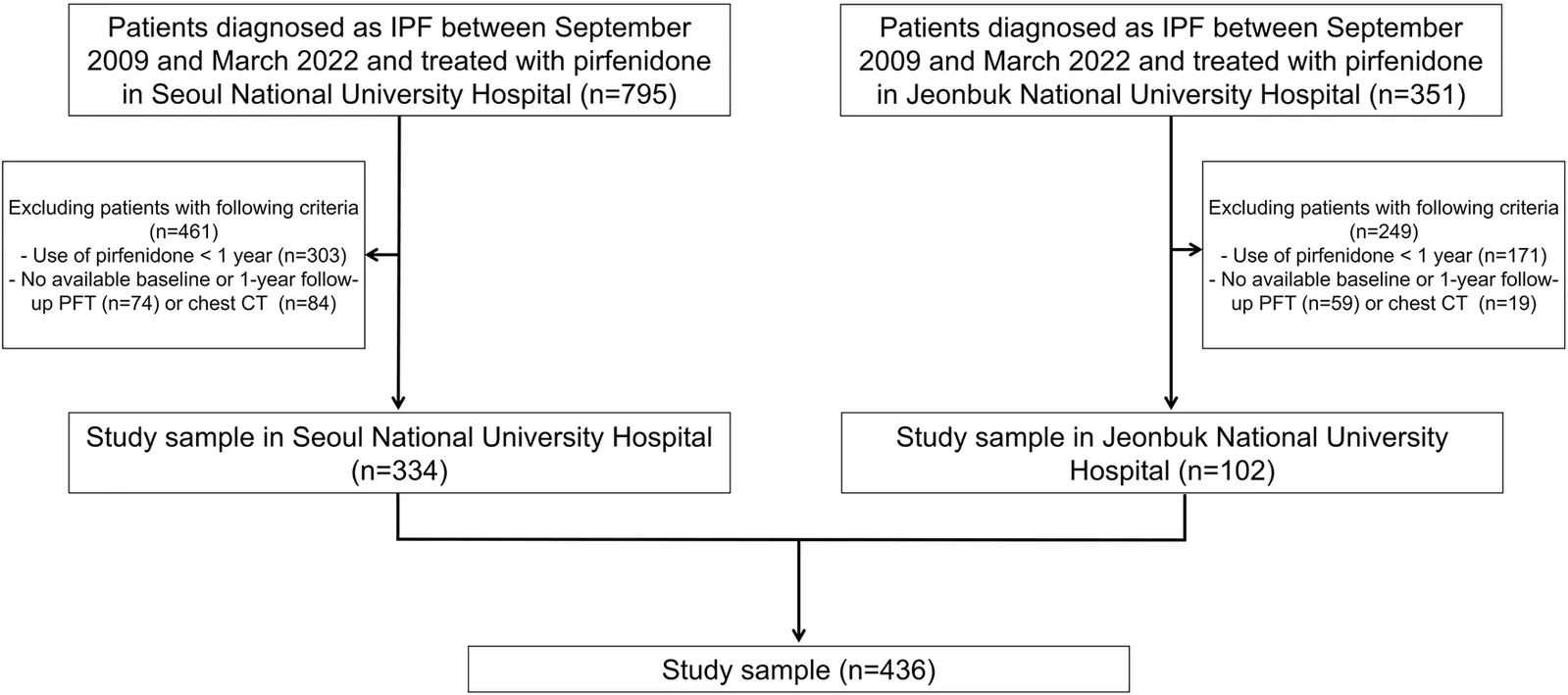
Flowchart of the study population. IPF, idiopathic pulmonary fibrosis; PFT, pulmonary function test

IPF was diagnosed through multidisciplinary team discussions at each center, integrating clinical, radiologic, and histologic data when available. We excluded IPF patients who met any of the following criteria: (1) use of pirfenidone for less than 1 year; (2) lack of baseline pulmonary function test (PFT) or chest CT scans at the time of pirfenidone administration or 1-year follow-up PFT. Baseline and 1-year follow-up PFTs and chest CT scans were defined with a buffer of 3 additional months to account for variability in patients’ follow-up schedules (i.e., examinations conducted within 3 months before and after starting pirfenidone were defined as baseline examinations; examinations conducted between 9 and 15 months after this time point were defined as 1-year follow-up examinations) (Fig. 1).

### CT acquisition

In this study, chest CT scans were acquired using 11 different scanners. The CT protocols were various in terms of detector row, contrast media usage, and the application of low-dose techniques, reconstruction kernels, and slice thickness. Detailed information regarding the CT acquisition is provided in the Supplementary Text and Supplementary Table S1.

Given that heterogeneity in CT acquisition could potentially impact segmentation results [27], we identified patients whose baseline and follow-up scans were acquired using imaging parameters consistent with those used during the development of the deep learning–based texture analysis software employed in this study: non-contrast usage, sharp-reconstruction kernels, and a slice thickness of 1.25 mm or less [28, 29]. This subset was utilized for the subgroup analysis described hereafter. Although lower detector-row scanners (e.g., 16-channel) may limit detailed fibrosis assessment, the software is not substantially affected by detector configuration; therefore, channel count was not considered in this categorization. Furthermore, because dose variation did not significantly correlate with CT quantification results, and to avoid further limiting the sample size, the inclusion of low-dose techniques was deemed acceptable [30]. Detailed information regarding CT acquisition is provided in the Supplementary Text.

### Clinical-radiological variables

Clinical data were collected from electronic medical records and included age, sex, body mass index (BMI), smoking status, the daily dose of pirfenidone, lung cancer history, and baseline and follow-up PFT results, including FVC (% predicted) and diffusing capacity of carbon monoxide (DL_CO_) (%). Changes in FVC (% predicted) after 1 year of antifibrotic treatment were defined as absolute changes, calculated as the follow-up value minus the baseline value. Radiologic data from both hospitals—including usual interstitial pneumonia (UIP) pattern classification and main pulmonary artery diameter measurements on baseline CT scans [31–34]—were independently evaluated by two thoracic radiologists (J.Y.L. and J.H.L., with 6 and 12 years of experience in thoracic imaging, respectively) for the purposes of this study, and any discrepancies were resolved by consensus (Supplementary Text).

### CT quantification

Commercially available deep learning–based texture analysis software (AVIEW Lung Texture version 1.1.44.27, Coreline Soft) was used for quantification of baseline and follow-up CT images (Supplementary Text). This analysis was performed by a radiologist (J.H.L.). The software performs fully automatic segmentation of the total lung parenchyma and each finding (normal lung parenchyma, ground-glass opacity (GGO), reticular opacity, honeycombing cyst, emphysema, and consolidation) on CT images, providing quantification results as a percentage of the total lung volume [27–29]. Because a primary objective of this study is to apply fully automated segmentation into real-world clinical practice, we utilized the segmentation results without any manual revision. In this study, fibrosis extent was defined as the sum of reticular opacity and honeycombing cysts, while ILD extent was defined as the combined sum of fibrosis and GGO [27, 35]; the extents of normal lung, fibrosis, and ILD were independently assessed (Supplementary Text). Changes in these CT quantification results after 1 year of antifibrotic treatment were calculated as the follow-up value minus the baseline value. There were no cases of segmentation failure in our study sample.

### Outcomes

The primary outcome was a composite of all-cause mortality or lung transplantation. Survival status and dates of death were obtained from a database maintained by the Ministry of the Interior and Safety, Korea, and information on lung transplantation was retrieved from electronic medical records. Survival time was censored as of October 31, 2023. For patients who died or underwent transplantation, the event time was defined as the date of death or the date of transplantation, respectively. Because this study focused on CT quantification in patients who received at least 1 year of antifibrotic treatment, early death or lung transplantation occurring within the first year could not be assessed.

The secondary outcome was a ≥ 10% absolute decline in FVC occurring after the first year of follow-up among patients who had not experienced this decline within the first year.

### Statistical analysis

Continuous variables are presented as mean ± standard deviation or median with interquartile range (IQR), depending on the normality of their distributions.

Inter-reader agreement for UIP pattern classification and main pulmonary artery diameter measurements was evaluated using weighted Cohen’s kappa and the intraclass correlation coefficient (ICC), respectively. To examine the relationship between changes in CT quantification and changes in FVC after 1 year of pirfenidone treatment, simple correlation analyses were performed.

To address potential selection bias—arising because the study sample was limited to patients receiving pirfenidone for at least 1 year and excluded those with shorter follow-up due to rapid clinical deterioration—we compared baseline characteristics, including age, sex, smoking status, BMI, and predicted FVC and DLCO (% predicted), between the included study sample and excluded groups using the Student’s *t*-test, Mann–Whitney U-test, or Pearson’s chi-square test, as appropriate. Additionally, differences in composite outcomes were evaluated using the log-rank test.

To assess the prognostic value of CT quantification results for the composite outcome, Cox proportional hazards regression analyses were conducted. Multivariable analyses were adjusted for clinico-radiologic covariates, including the gender–age–physiology (GAP) index—a well-established prognostic marker in IPF [36].

Additionally, two subgroup analyses were performed: (1) The aforementioned subgroup included patients whose baseline and follow-up CT scans were acquired using consistent imaging parameters; (2) Given the differences in clinical and radiologic characteristics between the two hospitals (Table 1), separate subgroup analyses were conducted for each hospital to evaluate the applicability of CT quantification across patients with varying disease profiles (e.g., disease severity reflected by baseline FVC).

**Table 1:** Baseline characteristics of the study sample

Variables	Study sample (*n* = 436)	Seoul National University Hospital (*n* = 334)	Jeonbuk National University Hospital (*n* = 102)	*p*-value^#^
Age (years)	69.8 ± 7.6	69.9 ± 7.7	69.5 ± 7.3	0.642
Sex				
Male	341 (78.2)	272 (81.4%)	69 (67.6%)	0.008
Female	95 (21.8)	62 (18.6%)	33 (32.4%)	
Baseline body mass index (kg/m^2^)	24.6 (22.9, 26.8)	24.5 (22.9, 26.8)	24.7 (22.9, 26.8)	0.870
Smoking status*				
Never smoker	144 (33.2)	98 (29.3%)	46 (46.0%)	0.002
Ever smoker	290 (66.8)	236 (70.7%)	54 (54.0%)	
Pirfenidone daily dose				
≥ 1200 mg	196 (45.0)	111 (33.2%)	85 (83.3%)	< 0.001
< 1200 mg	240 (55.0)	223 (66.8%)	17 (16.7%)	
Pirfenidone treatment period (months)	37.4 (24.8, 53.3)	34.8 (23.8, 48.3)	47.9 (29.1, 62.6)	< 0.001
History of lung cancer at baseline CT	41 (9.4)	38 (11.4%)	3 (2.9%)	0.010
Diagnosis of lung cancer after the baseline	23 (5.3)	13 (3.9%)	10 (9.8%)	0.002
Baseline FVC (% predicted)	79.0 (68.0, 90.0)	77.5 (67.0, 88.0)	83.5 (74.8, 96.0)	< 0.001
Baseline DL_CO_ (% predicted)	61.0 (49.0, 73.0)	61.0 (49.0, 72.0)	61.5 (46.8, 74.3)	0.940
Baseline GAP index	3 (3, 4)	3 (3, 4)	3 (2, 4)	< 0.001
Pulmonary artery diameter (mm)	29.0 (26.0, .32.0)	28.5 (26.0, 31.0)	30 (27.0, 33.0)	0.006
UIP pattern classification on CT				0.001
UIP pattern	243 (55.7)	202 (60.5%)	41 (40.2%)	
Probable UIP pattern	138 (31.7)	95 (28.4%)	43 (42.2%)	
Indeterminate for UIP pattern or alternative diagnosis	55 (12.6)	37 (11.1%)	18 (17.6%)	
Normal lung extent on baseline CT (%)	83.8 (76.4, 90.3)	83.3 (76.1, 89.1)	87.9 (77.7, 93.8)	0.002
Fibrosis extent on baseline CT (%)	11.1 (6.2, 17.7)	11.7 (7.4, 18.4)	6.8 (3.1, 15.4)	< 0.001
Change in normal lung extent after 1-year pirfenidone treatment (%)^†^	−1.3 (−6.2, 1.6)	−2.0 (−7.4, −1.3)	0.2 (−3.1, 2.4)	< 0.001
Change in fibrosis extent after 1-year pirfenidone treatment (%)^†^	1.4 (−0.9, 4.8)	1.7 (−0.8, 5.7)	0.5 (−1.1, 2.3)	0.004
Outcomes				
Primary outcome (composite outcome)	194 (44.5)	156 (46.7%)	38 (37.3%)	0.095
All-cause mortality	175 (40.1)	137 (41.0%)	38 (37.3%)	
Lung transplantation	19 (4.4)	19 (5.7%)	0 (0%)	
Follow-up duration (primary outcome) (months)	45.0 (33.0, 59.0)	43.0 (32.0, 58.0)	48.5 (34.0, 62.0)	-
≥ 10% FVC decline^‡^	186 (52.1)	149 (44.6%)	37 (36.2%)	0.134
Follow-up duration for FVC decline (months)^§^	22.0 (12.0, 39.5)	21.0 (11.0, 37.0)	28.5 (12.8, 47.0)	-

Data are reported as mean ± standard deviation, number (percentage), or median (interquartile range). The median intervals between pulmonary function tests and CT scans were 0 months (interquartile range, 0–1 months) at baseline and 1 month (interquartile range, 0–3 months) at the follow-up after 1-year pirfenidone treatment

*DLCO* diffusing capacity of carbon monoxide, *FVC* forced vital capacity, *GAP* gender–age–physiology, *UIP* unusual interstitial pneumonia

* Smoking status was missing for 2 patients

^†^ Median interval between baseline and follow-up CT images was 12 months (interquartile range: 11–14 months). In each hospital, median intervals were 12 months (interquartile range: 11–14 months) and 12 months (interquartile range: 11–13 months) at Seoul National University Hospital and Jeonbuk National University Hospital, respectively

^‡^ A decline in FVC of 10% after 1 year was assessed in 357 patients (Seoul National University Hospital, *n* = 275; Jeonbuk National University Hospital, *n* = 82)

^§^ This period represents the time between the follow-up CT scan and the censoring date for pulmonary function testing (i.e., the date of ≥ 10% FVC decline or the last available pulmonary function test)

^#^ Comparisons between Seoul National University Hospital and Jeonbuk National University Hospital, using the Student’s *t*-test or Mann–Whitney U-test for continuous variables and the Pearson’s chi-square test for categorical variables

A Cox proportional hazards regression was performed for the secondary outcome, defined as a ≥ 10% absolute decline in FVC following the follow-up CT scan. Patients who died or underwent lung transplantation prior to this outcome were censored at the date of their final PFT, as subsequent assessments were unavailable. To evaluate the robustness of these findings, a sensitivity analysis was conducted using Fine–Gray subdistribution hazard models, treating death or lung transplantation within 6 months of the final CT as competing risks; this 6-month interval was established because PFTs are clinically unfeasible at the immediate time of death or transplantation.

Finally, exploratory threshold-based analyses of changes in normal lung and fibrosis extent were performed to facilitate clinical identification of patients at high risk for the composite outcome. Kaplan–Meier survival curves with log-rank tests were used for comparison. Optimal integer thresholds were determined as those yielding the minimum *p*-value from the log-rank test for transplantation-free survival.

**Fig. 2:**
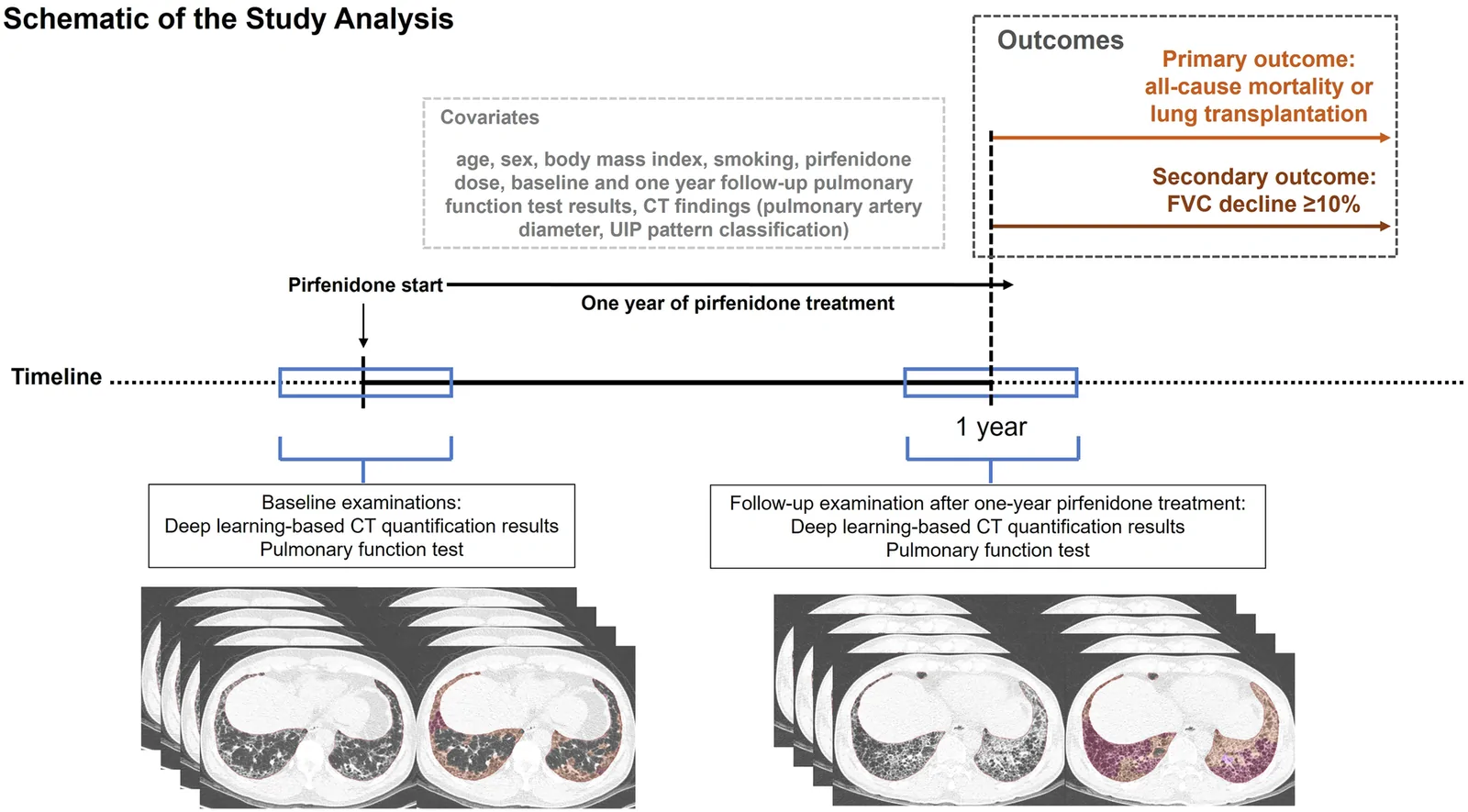
Analytical scheme of this study. This study evaluated the prognostic value of changes in deep learning–based CT quantification for predicting all-cause mortality or lung transplantation (primary outcome) and ≥ 10% FVC decline (secondary outcome) in patients with idiopathic pulmonary fibrosis who received at least 1 year of pirfenidone treatment. FVC, forced vital capacity; UIP, usual interstitial pneumonia

The analytical scheme is illustrated in Fig. 2. Detailed descriptions of the statistical comparison between the included and excluded patient groups, the radiologic evaluation for UIP pattern classification, measurements of the main pulmonary artery diameter, and the survival analyses are provided in the Supplementary Text. All statistical analyses were performed using SPSS version 29.0 (IBM Corp.), PASS 2022 (NCSS, LLC), and R version 4.2.1 (R Foundation for Statistical Computing). A two-sided *p*-value < 0.05 was considered statistically significant.

## Results

### Baseline characteristics

A total of 436 patients (341 men; mean age, 69.8 ± 7.6 years; 334 from Seoul National University Hospital and 102 from Jeonbuk National University Hospital) were included. They were administered pirfenidone for a median of 37.4 months (interquartile range (IQR), 24.8–53.3 months). Baseline CT scans showed a median normal lung extent of 83.8% (IQR, 76.4–90.3%) and a median fibrosis extent of 11.1% (IQR, 6.2–17.7%). After 1 year of pirfenidone treatment, follow-up CT scans demonstrated a median decrease of 1.3% in normal lung extent (IQR, −6.2% to 1.6%) and a median increase of 1.4% in fibrosis extent (IQR, −0.9% to 4.8%) (Table 1).

Over a median follow-up of 45.0 months (IQR, 33.0–59.0 months), 194 patients (44.5%) experienced the composite outcome (comprising all-cause mortality (*n* = 175, 40.1%) and lung transplantation (*n* = 19, 4.4%)). Lung transplantation was performed at a median of 46.0 months (IQR, 30.5–59.8 months) after baseline CT.

A total of 357 patients were eligible for assessing a ≥ 10% FVC decline after 1 year of pirfenidone therapy. During a median follow-up of 22.0 months after the follow-up CT scan (IQR, 12.0–39.5 months), 186 patients (52.1%) exhibited an FVC decline of ≥ 10%.

For UIP pattern classification, the weighted κ value was 0.567 (95% confidence interval (CI), 0.489, 0.645). For main pulmonary artery diameter measurements, the ICC was 0.900 (95% CI, 0.881, 0.917).

Detailed comparisons between the included study sample and excluded patients are provided in the Supplementary Text, Supplementary Table S2, and Supplementary Fig. S1.

### Relationship between CT quantification results and FVC after 1-year treatment

Changes in CT quantification results were significantly correlated with changes in FVC after 1-year pirfenidone treatment (normal lung extent: r = 0.325, 95% CI: 0.239, 0.407, *p* < 0.001; fibrosis extent: r = −0.370, 95% CI: −0.449, 0.286, *p* < 0.001; Supplementary Fig. S2).

### Prognostic value of CT quantification

**Fig. 3:**
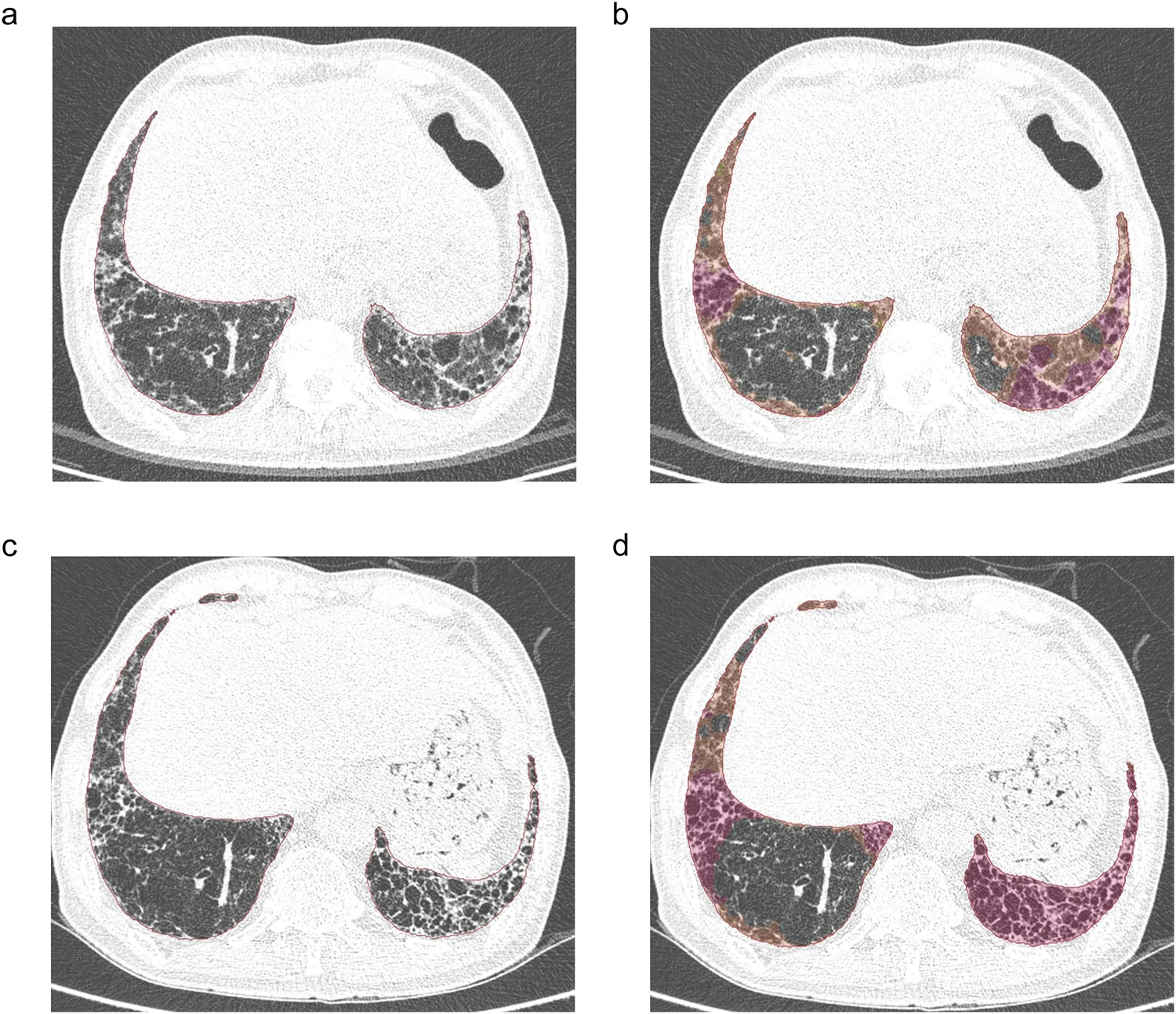
Representative case of prognostic value of change in CT quantification after 1-year pirfenidone treatment. **a** Unenhanced axial CT image obtained at the initiation of pirfenidone treatment in a 63-year-old man with idiopathic pulmonary fibrosis. **b** Segmentation overlays of the baseline CT images demonstrate reticular opacity (orange) and honeycombing cysts (red). The normal lung extent and fibrosis extent (reticular opacity plus honeycombing cysts) were 79.5% and 18.4%, respectively. **c** Unenhanced axial CT images obtained after 14 months of pirfenidone treatment. **d** Segmentation overlays of the follow-up CT images show a normal lung extent of 73.0% and a fibrosis extent of 24.8%, corresponding to changes of −6.5% and +6.5%, respectively. The patient died 59 months after initiation of pirfenidone treatment

In univariable analyses, changes in normal lung extent and fibrosis extent after 1-year pirfenidone treatment were associated with the composite outcome (both *p*-values < 0.001; Supplementary Table S3). In multivariable analyses, the prognostic value of these CT quantification changes persisted for both normal lung extent (adjusted hazard ratio (HR) per 5% change: 0.90, 95% CI: 0.85, 0.96, *p* < 0.001) and fibrosis extent (adjusted HR per 5% change: 1.16, 95% CI: 1.06, 1.27, *p* = 0.002) (Fig. 3 and Table 2). The results for ILD extent were consistent (adjusted HR per 5% change: 1.16, 95% CI: 1.08, 1.24, *p* < 0.001) (Supplementary Table S4).

**Table 2:** Multivariable Cox proportional hazards regression analyses for predicting the composite outcome (all-cause mortality or lung transplantation) in patients with IPF who completed at least 1 year of pirfenidone therapy

Normal lung extent		
Variables	Hazard ratio	*p*-value
GAP index	1.37 (1.17, 1.60)	< 0.001
Change in FVC after 1-year pirfenidone treatment (% predicted)	0.95 (0.93, 0.96)	< 0.001
Body mass index (kg/m^2^)	0.99 (0.95, 1.04)	0.679
Smoking (reference: never smoker)	1.13 (0.83, 1.55)	0.439
Pirfenidone daily dose ≥ 1200 mg (reference: < 1200 mg)	0.95 (0.70, 1.28)	0.718
Pulmonary artery diameter (mm)	1.03 (0.99, 1.06)	0.115
UIP pattern (reference: indeterminate UIP pattern or alternative diagnosis)		
Probable UIP pattern	1.16 (0.69, 1.96)	0.573
UIP pattern	1.26 (0.78, 2.03)	0.346
Normal lung extent on baseline CT (per 5%)	0.85 (0.79, 0.89)	< 0.001
Change in normal lung extent after 1-year pirfenidone treatment (per 5%)	0.90 (0.85, 0.96)	< 0.001

Fibrosis extent		
Variables	Hazard ratio	*p*-value
GAP index	1.42 (1.22, 1.65)	< 0.001
Change in FVC after 1-year pirfenidone treatment (% predicted)	0.95 (0.93, 0.96)	< 0.001
Body mass index (kg/m^2^)	0.98 (0.94, 1.03)	0.430
Smoking (reference: never smoker)	1.20 (0.88, 1.65)	0.243
Pirfenidone daily dose ≥ 1200 mg (reference: < 1200 mg)	1.04 (0.78, 1.40)	0.774
Pulmonary artery diameter (mm)	1.03 (0.99, 1.06)	0.163
UIP pattern (reference: indeterminate UIP pattern or alternative diagnosis)		
Probable UIP pattern	0.95 (0.57, 1.60)	0.857
UIP pattern	0.96 (0.59, 1.56)	0.870
Fibrosis extent on baseline CT (per 5%)	1.25 (1.15, 1.34)	< 0.001
Change in fibrosis extent after 1-year pirfenidone treatment (per 5%)	1.16 (1.06, 1.27)	0.002

*DLCO* diffusing capacity of carbon monoxide, *FVC* forced vital capacity, *GAP* gender–age–physiology, *IPF* idiopathic pulmonary fibrosis, *UIP* unusual interstitial pneumonia

A subgroup analysis in patients whose CT scans were acquired under consistent imaging parameters at baseline and follow-up showed similar results, although the change in normal lung extent after 1-year treatment did not reach statistical significance (Supplementary Table S5). Across hospitals, the CT quantification changes were consistent risk factors, except that the change in fibrosis extent after 1-year treatment was not significant in Jeonbuk National University Hospital (Supplementary Table S6 and Supplementary Text).

### FVC decline after 1-year pirfenidone treatment

In univariable analyses, the changes in normal lung extent and fibrosis extent after 1-year pirfenidone treatment were predictive of ≥ 10% FVC decline (both *p*-values < 0.001; Supplementary Table S2). After adjusting clinic-radiological factors, the change in fibrosis extent remained a risk factor for ≥ 10% FVC decline (adjusted HR per 5% change: 1.26, 95% CI: 1.09, 1.45, *p* = 0.001), whereas the change in normal lung extent demonstrated only borderline significance (adjusted HR per 5% change: 0.93, 95% CI: 0.85, 1.01, *p* = 0.059) (Table 3). Change in fibrosis extent was a consistent predictor in the competing risk analysis (adjusted subdistribution HR per 5% change: 1.20, 95% CI: 1.04, 1.40; *p* = 0.016; Table 4). Corresponding results for ILD extent are detailed in Supplementary Table S4.

**Table 3:** Multivariable Cox proportional hazards regression analyses for predicting an FVC decline of ≥ 10% in patients with IPF who completed at least 1 year of pirfenidone therapy

Normal lung extent		
Variables	Hazard ratio	*p*-value
Age	1.02 (1.00, 1.04)	0.033
Sex (reference: female)	0.68 (0.42, 1.10)	0.116
Body mass index (kg/m^2^)	1.05 (1.00, 1.11)	0.063
Smoking (reference: never smoker)	0.68 (0.46, 1.00)	0.051
Pirfenidone daily dose ≥ 1200 mg (reference: < 1200 mg)	0.92 (0.67, 1.25)	0.576
Baseline FVC (% predicted)	1.01 (1.00, 1.02)	0.202
Baseline DLco (% predicted)	1.00 (0.99, 1.01)	0.691
Change in FVC after 1-year pirfenidone treatment (% predicted)	0.89 (0.86, 0.92)	< 0.001
Pulmonary artery diameter (mm)	0.97 (0.94, 1.01)	0.206
UIP pattern (reference: indeterminate UIP pattern or alternative diagnosis)		
Probable UIP pattern	1.56 (0.93, 2.62)	0.089
UIP pattern	1.43 (0.88, 2.32)	0.153
Normal lung extent on baseline CT (per 5%)	0.96 (0.87, 1.06)	0.391
Change in normal lung extent after 1-year pirfenidone treatment (per 5%)	0.93 (0.85, 1.01)	0.059

Fibrosis extent		
Variables	Hazard ratio	*p*-value
Age	1.02 (1.00, 1.05)	0.027
Sex (reference: female)	0.66 (0.41, 1.05)	0.081
Body mass index (kg/m^2^)	1.04 (0.99, 1.10)	0.104
Smoking (reference: never smoker)	0.71 (0.49, 1.04)	0.082
Pirfenidone daily dose ≥ 1200 mg (reference: < 1200 mg)	0.94 (0.69, 1.28)	0.700
Baseline FVC (% predicted)	1.01 (1.00, 1.02)	0.095
Baseline DLco (% predicted)	1.00 (0.99, 1.01)	0.513
Change in FVC after 1-year pirfenidone treatment (% predicted)	0.89 (0.86, 0.92)	< 0.001
Pulmonary artery diameter (mm)	0.97 (0.93, 1.01)	0.094
UIP pattern (reference: indeterminate UIP pattern or alternative diagnosis)		
Probable UIP pattern	1.59 (0.96, 2.65)	0.073
UIP pattern	1.39 (0.84, 2.31)	0.201
Fibrosis extent on baseline CT (per 5%)	1.07 (0.95, 1.20)	0.277
Change in fibrosis extent after 1-year pirfenidone treatment (per 5%)	1.26 (1.09, 1.46)	0.001

*DLCO* diffusing capacity of carbon monoxide, *FVC* forced vital capacity, *GAP* gender–age–physiology, *IPF* idiopathic pulmonary fibrosis, *UIP* unusual interstitial pneumonia

**Table 4:** Multivariable Fine–Gray proportional regression analyses of FVC decline ≥ 10% considering death or lung transplantation as competing risks in patients with IPF after 1 year of pirfenidone therapy

Normal lung extent		
Variables	sHR	*p*-value
Age	1.01 (0.99, 1.03)	0.310
Sex (reference: female)	1.53 (0.93, 2.51)	0.091
Body mass index (kg/m^2^)	1.06 (1.01, 1.11)	0.019
Smoking (reference: never smoker)	0.61 (0.42, 0.90)	0.012
Pirfenidone daily dose ≥ 1200 mg (reference: < 1200 mg)	0.92 (0.68, 1.26)	0.610
Baseline FVC (% predicted)	1.01 (1.00, 1.02)	0.130
Baseline DLco (% predicted)	1.00 (0.99, 1.01)	0.660
Change in FVC after 1-year pirfenidone treatment (% predicted)	0.89 (0.86, 0.92)	< 0.001
Pulmonary artery diameter (mm)	0.96 (0.93, 1.00)	0.033
UIP pattern (reference: indeterminate UIP pattern or alternative diagnosis)		
Probable UIP pattern	1.41 (0.88, 2.27)	0.160
UIP pattern	1.59 (0.99, 2.55)	0.055
Normal lung extent on baseline CT (per 5%)	0.97 (0.88, 1.07)	0.540
Change in normal lung extent after 1-year pirfenidone treatment (per 5%)	0.94 (0.86, 1.03)	0.180

Fibrosis extent		
Variables	sHR	*p*-value
Age	1.01 (0.99, 1.03)	0.290
Sex (reference: female)	1.55 (0.93, 2.60)	0.093
Body mass index (kg/m^2^)	1.06 (1.00, 1.11)	0.033
Smoking (reference: never smoker)	0.64 (0.43, 0.94)	0.023
Pirfenidone daily dose ≥ 1200 mg (reference: < 1200 mg)	0.95 (0.69, 1.29)	0.720
Baseline FVC (% predicted)	1.01 (1.00, 1.02)	0.064
Baseline DLco (% predicted)	1.00 (0.99, 1.01)	0.540
Change in FVC after 1-year pirfenidone treatment (% predicted)	0.89 (0.86, 0.92)	< 0.001
Pulmonary artery diameter (mm)	0.95 (0.92, 0.99)	0.014
UIP pattern (reference: indeterminate UIP pattern or alternative diagnosis)	1.37 (0.82, 2.30)	0.230
Probable UIP pattern	1.61 (0.98, 2.64)	0.059
UIP pattern		
Fibrosis extent on baseline CT (per 5%)	1.05 (0.93, 1.19)	0.440
Change in fibrosis extent after 1-year pirfenidone treatment (per 5%)	1.20 (1.04, 1.40)	0.016

*DLCO* diffusing capacity of carbon monoxide, *FVC* forced vital capacity, *GAP* gender–age–physiology, *IPF* idiopathic pulmonary fibrosis, *sHR* subdistribution hazard ratio, *UIP* unusual interstitial pneumonia

### Threshold-based analysis

**Fig. 4:**
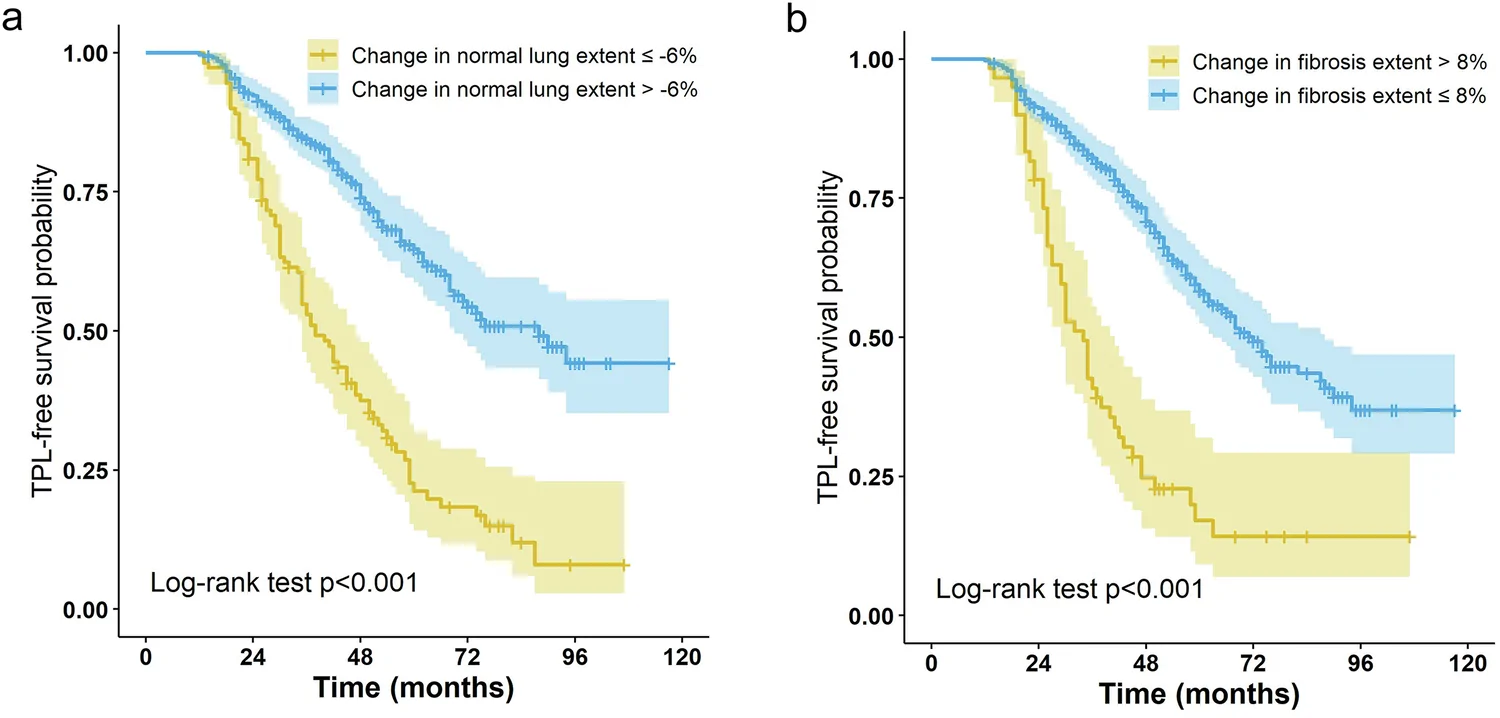
Kaplan–Meier plots for the composite outcome (all-cause mortality or lung transplantation), stratified by changes in normal lung and fibrosis extents after 1-year pirfenidone treatment. **a** Patients with a ≥ 6% decrease in normal lung extent experienced the composite outcomes more frequently than those without such a decrease (78.2% (86/110) versus. 33.1% (108/326), *p* < 0.001). **b** Patients with an ≥ 8% increase in fibrosis extent had a higher frequency of the composite outcomes than those who did not (80.0% (48/60) versus 38.8% (146/376), *p* < 0.001). Shaded areas denote 95% confidence intervals, and ticks indicate censored patients

The optimal thresholds for transplantation-free survival were a 6% decrease in normal lung extent and an 8% increase in fibrosis extent after 1-year pirfenidone treatment. Specifically, patients with a ≥ 6% decrease in normal lung extent experienced the composite outcome more frequently than those without such a decrease (78.2% (86 of 110) versus 33.1% (108 of 326), *p* < 0.001). And patients with an ≥ 8% increase in fibrosis extent had a higher frequency of these events than those who did not (80.0% (48 of 60) vs. 38.8% (146 of 376), *p* < 0.001) (Fig. 4).

## Discussion

Despite the proven efficacy of antifibrotic agents in slowing lung function decline in patients with IPF, disease progression and mortality continue to occur unpredictably beyond 1 year of treatment—outside the landmark periods typically evaluated in major clinical trials [9–12]. To address this gap, we evaluated the prognostic value of changes in deep learning–based CT quantification of normal lung and fibrosis extents in patients with IPF who completed at least 1 year of pirfenidone therapy. Greater preservation of normal lung extent over the 1-year treatment was associated with a lower risk of the composite outcome (adjusted HR per 5% change: 0.90), whereas increases in fibrosis extent were associated with a higher risk (adjusted HR per 5% change: 1.16). In addition, changes in fibrosis extent also predicted ≥ 10% FVC decline (adjusted HR per 5% change: 1.26; adjusted subdistribution HR per 5%: 1.20). The prognostic value of ILD extent—comprising both fibrosis and GGO—was consistent (HR for composite outcome per 5% increase: 1.16; HR for FVC decline ≥ 10% per 5% increase: 1.22).

Recent studies have applied deep learning–based prognostication in IPF; however, their cohorts were not limited to patients receiving antifibrotic therapy—that is, they included both antifibrotic-treated and untreated patients. The key findings and distinctions from our study are summarized as follows: Devaraj et al reported that a weighted reticulovascular score derived from CT scans could predict FVC decline at 52 weeks in patients with IPF, although the covariates were not clearly specified [18]. This score was also proposed as a prognostic factor for a ≥ 10% FVC decline and mortality in patients with IPF [19, 20]. Thillai et al reported that lung and fibrosis volumes segmented by deep learning algorithms were prognostic for mortality in patients with IPF [21]. Although their analysis included both baseline and follow-up CT scans, the timing of follow-up imaging varied widely, ranging from 6 to 24 months after baseline [21]. Koslow et al demonstrated that a 1-year change in fibrosis quantification could predict FVC decline and transplant-free survival in patients with fibrotic ILD, including IPF [37]; however, their study did not evaluate changes in normal lung extent. Notably, these longitudinal studies did not adjust for FVC change, a known predictor of disease progression and mortality in IPF [4]. Beyond fibrosis quantification, Humphries et al reported the prognostic significance of deep learning–based CT UIP classification [22]. Unlike prior studies, our analysis specifically targets patients who completed at least 1 year of pirfenidone therapy, enhancing the validity and applicability of deep learning–based quantification of normal lung and fibrosis extents as prognostic indicators in this population.

It is noteworthy that the change in normal lung extent after 1-year pirfenidone treatment was associated with the composite outcome. We believe this reflects the physiological relationship whereby survival depends on the preservation of functional lung parenchyma, represented by the normal lung extent after excluding emphysema, consolidation, ground-glass opacity, and fibrosis. This observation is consistent with previous studies demonstrating the prognostic value of normal lung extent in patients with IPF—including those not treated with antifibrotic agents—as well as in idiopathic pleuroparenchymal fibroelastosis and systemic sclerosis–related ILD [38–42]. In contrast, FVC decline from a given time point may be more directly driven by changes in non-functional lung tissue (i.e., fibrosis extent) at that same time point. Supporting this interpretation, the increase in fibrosis extent after 1-year pirfenidone treatment was predictive of ≥ 10% FVC decline and may contribute to the subsequent composite outcome in our study [37].

Although baseline normal lung and fibrosis extents were identified as risk factors—consistent with prior reports [43]—we further demonstrated that their 1-year longitudinal changes were significant predictors of the composite outcome, even after adjusting for baseline values. These findings suggest that temporal changes in CT quantification provide incremental prognostic information beyond static measurements by capturing the patient’s evolving disease status.

Moderate interobserver agreement for UIP pattern classification on CT (κ = 0.567) was demonstrated in our study, which is consistent with prior studies showing κ values ranging from 0.48 to 0.52 and a pooled κ of 0.61 [26, 44]. Although the presence of honeycombing is an essential criterion for diagnosing a UIP pattern on CT [31], interobserver agreement for identifying honeycombing itself is also moderate, with κ values ranging from 0.40 to 0.58 [25]. Moreover, UIP pattern categorization requires integrating multiple imaging features—not only the presence of honeycombing but also its distribution (e.g., basal and subpleural predominance) and its relationship to other findings such as the extent of ground-glass opacity—further contributing to variability among radiologists. Considering that the ultimate goal of UIP CT categorization is to enhance prognostication and guide management strategies [31], a fully automated CT quantification approach—as demonstrated by its prognostic value in this study—effectively supports this objective by overcoming the inherent challenge of interobserver variability.

Several limitations of this study should be noted. First, while the heterogeneity in CT acquisition protocols reflects real-world practice and supports the generalizability of our findings, such variability may have influenced the quantification metrics [27]. Although subgroup analyses were performed to mitigate this impact, the reduced sample size (*n* = 241) partially limited their interpretation. Therefore, further validation in larger cohorts using protocols consistent with the model’s development dataset is warranted. Second, the limited sample sizes in subgroup analyses constrained the interpretation of the results; for instance, the change in fibrosis extent did not reach statistical significance at Jeonbuk National University Hospital. Third, although the extent of emphysema is strongly correlated with lung function decline and poor survival in IPF patients [45–47], it was not accounted for in the current study since the optimal CT acquisition and reconstruction parameters for emphysema quantification significantly differ from those required for ILD assessment [48]. The inclusion of emphysema quantification under CT conditions optimized for ILD could compromise automated segmentation performance, potentially yielding misleading results. Fourth, follow-up assessments, including PFTs, were basically performed at 3-month intervals in this study; however, these intervals could vary according to clinicians’ follow-up strategies and patients’ unexpected events. As a result, detection bias may have been introduced in the secondary outcome analysis. Fifth, we did not evaluate acute exacerbations of IPF as an outcome. Sixth, we did not perform a reproducibility test of the CT quantification software on our study sample; however, this software has demonstrated high reproducibility in quantifying fibrosis (e.g., variability < 1%) [27]. Finally, as the threshold-based analysis yielded preliminary results derived from a single cohort, further validation in independent, prospective cohorts is essential before clinical application.

In conclusion, deep learning–based CT quantification changes after 1 year of pirfenidone treatment demonstrated prognostic value in patients with IPF, predicting all-cause mortality or lung transplantation as well as subsequent lung function decline.

## Supplementary information

ELECTRONIC SUPPLEMENTARY MATERIAL

## Data Availability

The datasets generated or analyzed during the study are available from the corresponding author on reasonable request.

## Abbreviations

FVC: Forced vital capacity
ICC: Intraclass correlation coefficient
IPF: Idiopathic pulmonary fibrosis
IQR: Interquartile range
PFT: Pulmonary function test
UIP: Usual interstitial pneumonia
